# Remission of long-term conditions among adults with multiple long-term conditions in England primary care: A retrospective cohort study of 4.7 million people

**DOI:** 10.1186/s12916-026-05106-1

**Published:** 2026-08-10

**Authors:** Aman Jat, Benjamin D Barton, Rohini Mathur, Beth Stuart, Hilda Hounkpatin, Hajira Dambha-Miller

**Affiliations:** 1 Primary Care Research Centre, University of Southampton, Southampton, SO16 5ST United Kingdom; 2 Wolfson Institute of Population Health, Queen Mary University of London, London, United Kingdom

**Keywords:** Multimorbidity, Remission, Electronic health records, Primary care

## Abstract

**Background:**

Multiple long-term conditions (MLTC) are increasingly prevalent among UK adults, yet the extent to which remission of long-term conditions is recorded in routine primary care remains poorly understood. Accurate documentation of remission has implications for clinical management, disease trajectories, and the use of electronic health records in research. Examining remission in the context of MLTC is important because remission in one condition may alter overall disease burden and treatment needs in people living with several coexisting conditions. We aimed to describe the frequency and variation of recorded remission across multiple long-term conditions among adults.

**Methods:**

We conducted a retrospective cohort study using linked primary care, hospital, and CPRD Aurum data. Adults aged ≥ 18 with MLTC (≥ 2 long-term conditions) between Jan 1, 1987, and Dec 31, 2020, were included. Remission was defined using condition-specific criteria based on remission or resolution codes, relevant clinical measurements meeting predefined thresholds, treatment patterns, and condition-specific clinical events recorded in electronic health records. We examined remission for 10 conditions with operationalizable definitions. Analyses were descriptive, summarising remission frequencies and sociodemographic patterns.

**Results:**

Among 4,745,099 adults followed for a median of 8 years, 900,609 (19%) had recorded remission of at least one condition. Remission varied substantially by condition, with the highest rates observed for anaemia (63.0%), asthma (44.71%), and diabetes (15.51%), calculated among people with an ever-recorded diagnosis of each condition. Those with remission were slightly younger and more often male; Asian and Black ethnic groups were under-represented, while deprivation patterns were broadly similar.

**Conclusions:**

Recorded remission in MLTC is highly heterogeneous and often rare. Variation between conditions and low rates of sustained remission likely reflect coding practices and clinical recording behaviours. Standardised remission definitions and consistent Systematised Nomenclature of Medicine (SNOMED) coding could improve data quality and support more reliable research on remission and disease trajectories.

**Supplementary Information:**

The online version contains supplementary material available at https://doi.org/10.1186/s12916-026-05106-1.

## Background

Multiple long-term conditions (MLTC), defined as the coexistence of two or more long-term conditions, is an increasing public health issue [1, 2]. Multimorbidity is common in England and is expected to increase as the population ages, with projections showing a substantial rise in the number of older adults living with multiple long-term conditions by 2035 [3–6]. MLTC is linked with poorer health, reduced mobility, lower quality of life, premature mortality, and higher use of health care [1, 2, 7–9]. It has been identified as a priority for global health research [3]. Primary care delivers much of the routine management for people with MLTC in the UK, and descriptive evidence on remission could inform how conditions are recorded, monitored, and understood [9, 10]. Most previous research has focused on the development and progression of MLTC, including the accumulation of conditions, inequalities in onset, and healthcare use [11–13]. From a life-course epidemiological perspective, diagnoses may remain meaningful even if a condition is no longer actively coded or treated, as prior disease contributes to cumulative risk and aetiology [11, 14, 15]. However, in routine electronic health records and clinical practice, disease status is also represented dynamically, with conditions recorded as active, resolved, or in remission [10, 15]. Understanding how remission is documented is therefore important for interpreting current disease burden, healthcare utilisation, and MLTC trajectories in real-world data [10]. Without accounting for remission, estimates of MLTC prevalence and complexity based solely on ever-recorded diagnoses may overstate current care needs and obscure changes in disease status over time [10].

Earlier evidence shows that remission of individual conditions is possible [16, 17]. For example, remission of diabetes has been reported after weight loss through lifestyle change or bariatric surgery [16, 17]. A scoping review of 178 studies identified 96 different definitions of remission in diabetes, illustrating both the potential for remission and variation in measurement [17]. Other studies have described remission of hypertension, depression, chronic kidney disease, and substance use disorders [18, 19]. These investigations, however, have mainly focused on single-condition populations. In routine UK primary care, resolved or remission codes are also applied to several conditions [15, 18]. For example, “resolved asthma” is coded in some patients and is associated with subsequent variation in respiratory outcomes [10]. In UK primary care, remission or resolution codes are used for some long-term conditions, although their application varies by condition and coding practice [10, 15]. These coding practices indicate that remission is recognised in clinical data, although criteria and follow-up vary [15, 17]. Only a small number of studies have considered remission in people with MLTC [13, 15]. In one London borough, applying remission and resolution codes to primary care records reduced estimates of MLTC prevalence by 2.2% [10, 15]. A cohort study of more than 800,000 adults in South London found that 1.9% experienced remission of at least one condition during 15 years of follow-up. Studies in other contexts, such as after bariatric surgery, have also shown remission across several conditions, but these were not designed to assess MLTC specifically [18, 20]. Reviews in asthma and diabetes similarly highlight the diversity of remission definitions across conditions and settings [10, 17]. Most research on MLTC has focused on the accumulation and coexistence of conditions, typically treating diagnoses as persistent once recorded [4, 12, 14, 21]. However, in routine clinical practice and electronic health records, some conditions may enter remission or be recorded as resolved [10, 13, 15]. Failure to account for remission may therefore lead to overestimation of current disease burden, misclassification of MLTC status, and a limited understanding of how disease profiles change over time [10, 15, 22]. Despite these implications, remission of individual long-term conditions within the context of MLTC has received relatively little attention [10, 13, 15].

Understanding how remission is captured in routine data, and how it varies across conditions, is important for interpreting electronic health records, refining estimates of MLTC prevalence, and informing clinical management, where treatment burden and monitoring needs depend on current rather than historical disease status [10, 15, 22]. In people living with MLTC, remission of a single condition may also alter the overall disease burden, treatment complexity, and polypharmacy, and affect how individuals are classified and managed within MLTC frameworks [7, 9, 15, 22]. Studying remission in this context, therefore, provides insights that cannot be obtained from single-condition populations [8, 10, 13, 15]. We aimed to describe the frequency and patterns of recorded remission of selected long-term conditions among adults with MLTC, and to examine how remission is represented in routine data and may influence estimates of disease burden and care needs.

## Methods

### Study design, population, and data sources

We undertook a descriptive study using data from the Clinical Practice Research Datalink (CPRD) Aurum [23]. CPRD Aurum contains anonymised, routinely collected primary care records from general practices in England, linked to Hospital Episode Statistics (HES), the English Index of Multiple Deprivation (IMD), national disease registries, and death registrations from the Office for National Statistics (ONS) [22, 24]. The database includes records from about 19 million patients and is broadly representative of the English population by age, sex, and socioeconomic position [18]. The study population comprised adults (≥ 18 years) with MLTC, registered with a CPRD-contributing practice at any time between 1 January 1987 and 31 December 2020. Entry was defined as the latest of: 1 January 1987, date of practice registration, 18th birthday, or date of diagnosis of a second long-term condition. Follow-up ended at the earliest of 31 December 2020, end of registration, or death. Patients were excluded if they lacked linked HES data, follow-up data, or had missing age, sex, ethnicity, or IMD.

### Definition of MLTC

MLTC was defined as the presence of two or more long-term conditions from a list of 59 long-term conditions, out of which 54 were identified in CPRD - developed through national consensus methods [25]. Conditions were identified using diagnostic codes in primary and secondary care records, supported where relevant by biochemical results, clinical assessments, or validated rating scales [23, 25]. For conditions with records in both primary and secondary care, the earliest diagnosis date was used. Absence of codes or test results was considered absence of the condition. The full list of long-term conditions used to define MLTC, corresponding code lists, and the operational definitions used to identify recorded remission for the 10 analysed conditions are provided in the Supplementary Material.

### Definition of remission

This study focused on describing remission in conditions for which operational definitions could be reliably applied within England’s electronic health records. Accordingly, remission in this study refers to recorded remission derived from available electronic health record data, rather than independently verified clinical remission. As remission could not be consistently defined for all long-term conditions, analyses were restricted to a subset of conditions with sufficient coding, test, or treatment data. We examined remission for 10 long-term conditions [10]. These conditions were identified through a structured process that combined a scoping review of remission definitions in electronic health records with consultation with clinical and data experts. This process was used to identify conditions for which remission was both clinically plausible and operationalizable using available primary care electronic health record data, including diagnostic or remission codes, laboratory results, treatment records, and condition-specific clinical events. Although MLTC was defined using 54 long-term conditions, not all conditions were considered amenable to remission, and robust EHR-based remission definitions could only be developed for 10 conditions [10, 25].

Remission was defined by evidence of any of the following: a diagnostic code for remission or resolution, test results or scores within accepted thresholds, clinical assessments consistent with remission, absence of condition-specific events (such as hospital admissions), or discontinuation of relevant treatment. Definitions were developed using a structured framework for identifying remission in England primary care electronic health records, informed by a scoping review of previous studies, consultation with clinicians, and review of published code lists [10]. Full operational definitions for each condition are provided in the appendix.

Follow-up time was calculated individually for each participant from the earliest date of eligibility to the end of registration, death, or study end. Because conditions varied in their typical age of onset and timing of first recorded diagnosis, available follow-up time differed across conditions.

### Handling of missing data

Remission was operationalised as a binary outcome based on predefined condition-specific criteria derived from diagnostic codes, clinical measurements, and treatment records. Individuals were classified as having remission only when these criteria were met. Participants without sufficient evidence to meet remission criteria were classified as having no recorded remission, rather than being excluded from analyses. This reflects the nature of routinely collected electronic health records, where absence of qualifying codes or measurements indicates no documented remission rather than missing outcome data.

Missing data in sociodemographic variables (age, sex, ethnicity, and Index of Multiple Deprivation) were assessed at cohort entry; individuals with missing values were excluded prior to analysis.

### Statistical analysis

We used descriptive statistics to summarise the characteristics of the study population, overall and stratified by remission status (defined as remission of one or more long-term conditions at any point during follow-up). Frequencies and proportions were reported for categorical variables and means with standard deviations or mean for continuous variables. For each of the 10 conditions, we calculated the number and proportion of individuals with the condition who experienced remission during follow-up. The denominator was the number of individuals with the condition recorded in their health record, and the numerator was the number of those individuals with evidence of remission. Conditions were then ranked by the proportion of people in remission to identify which conditions most commonly went into remission. For baseline prevalence, we used the number of individuals with each long-term condition recorded at cohort entry, defined as the latest of the 18th birthday, practice registration, or date of diagnosis of the second condition. For remission analyses, we used “ever-record” denominators, defined as the number of individuals who had a Read/SNOMED code for the condition at any point during follow-up. We also described patterns of remission by examining: (i) whether remission occurred in one condition only or in multiple conditions within the same individual, and (ii) variation in remission across sociodemographic groups (age, sex, ethnicity, and socioeconomic position). Age was summarised using means and standard deviations, sex and ethnicity were presented as proportions, and socioeconomic position was described using the Index of Multiple Deprivation quintiles. Remission was assigned only when diagnostic codes, clinical measures, or treatment records met predefined criteria; individuals without sufficient information were classified as having no recorded remission rather than being excluded from analyses. All analyses were conducted using Stata SE 19.5.

## Results

**Fig. 1:**
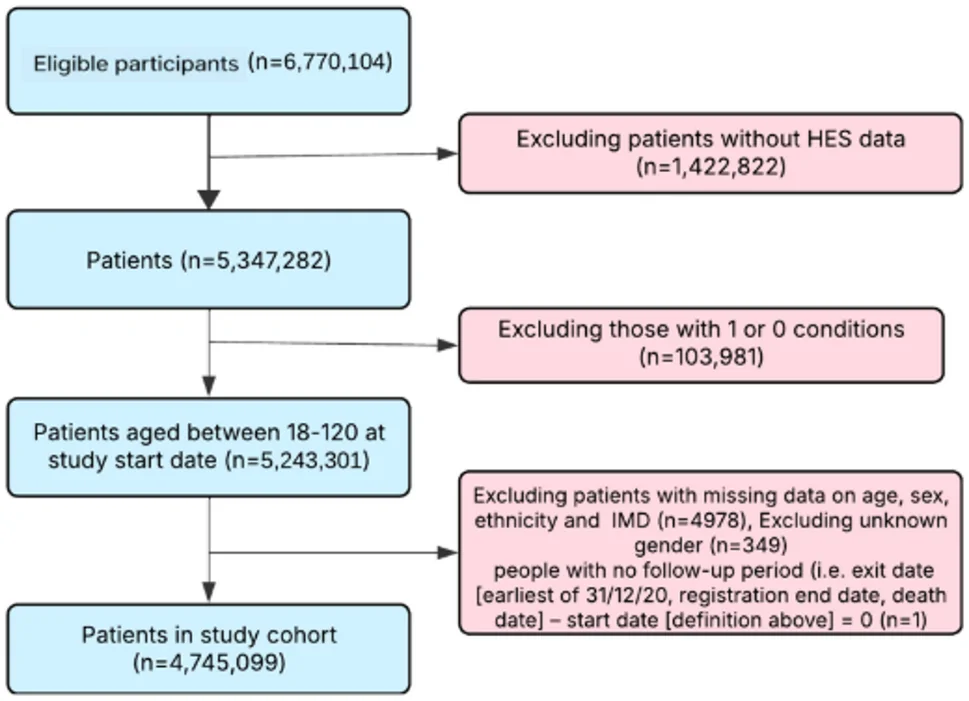
Participant flow diagram showing eligibility, exclusions, and final study cohort

Our study cohort included 4,745,099 adults aged 18 years or older with MLTC at baseline. Figure 1 presents the flow of participants through the study. The mean age was 58 years, 2,644,607 (55.73%) were female, and 4,351,881 (91.71%) identified as White ethnicity (Table 1). The mean number of long-term conditions at baseline was 2.50 (SD 1.44). The most prevalent conditions at baseline were hypertension (1,428,522; 30.11%), depression (1,128,140; 23.77%), and inflammatory bowel disease (838,552; 17.67%). Baseline characteristics are presented in Table 1.

**Table 1:** Baseline Characteristics of Study Participants in the CPRD MLTC Cohort: Comprehensive Sociodemographic, Clinical, and Long-Term Condition Profiles of 4,745,099 Adults Aged 18 Years and Over Contributing England Primary Care Data Between 1987 and 2020

Baseline characteristic	All (*n* = 4,745,099)
Sociodemographic variables	
Age, years (mean, SD)	58 (18.11)
18–40	870,716 (18.35%)
40–60	1,518,071 (31.99%)
60–80	1,847,204 (38.93%)
80–120	509,108 (10.73%)
Sex (n, %)	
Male	2,100,492 (44.27%)
Female	2,644,607 (55.73%)
Ethnicity	
White	4,351,881 (91.71%)
Asian	188,566 (3.97%)
Black	118,351 (2.49%)
Mixed	27,210 (0.57%)
Other	59,091 (1.25%)
Index of Multiple Deprivation (IMD) quintiles	
1 (least deprived)	898,699 (18.94%)
2	950,865 (20.04%)
3	913,092 (19.24%)
4	960,811 (20.25%)
5 (most deprived)	1,021,632 (21.53%)
**Clinical variables**	
Number of long-term conditions (LTCs)	
2 LTCs	3,310,253 (69.76%)
3-5LTCs	1,185,800 (24.99%)
6–50 LTCs	249,046 (5.25%)
LTCs median (IQR)	2 (2–3)
Has anaemia	96,859 (2.04%)
Has alcohol dependency	63,422 (1.34%)
Has asthma	556,443 (11.73%)
Has chronic kidney disease	116,225 (2.45%)
Has depression	1,128,140 (23.77%)
Has diabetes	497,623 (10.49%)
Has epilepsy	211,102 (4.45%)
Has hypertension	1,428,522 (30.11%)
Has heart failure	308,428 (6.50%)
Has inflammatory bowel disease	838,552 (17.67%)

N or n= Total population; Mean (SD); n (%)

During follow-up, 900 609 individuals (18.1%) had remission of at least one long-term condition, over a median follow-up of 8.0 years. Those with remission were marginally younger at baseline and more often male than those without remission and were less commonly from Asian or Black ethnic groups. The distribution of deprivation quintiles was broadly similar between groups (Table 3).

Marked variation in remission was observed across conditions (Table 2). The highest proportions of remission for at least 6 months were recorded for anaemia (560,445; 63.0%), asthma (676,291; 44.71%), and diabetes (301,313; 15.51%). Remission was uncommon in hypertension (30 124; 1.20%) and alcohol dependency (1 328; 0.25%) and was rare in chronic kidney disease (1 460; 0.14%), heart failure (1 273; 0.11%), and inflammatory bowel disease, where remission was recorded only in isolated cases. No remission events were observed for epilepsy, consistent with clinical expectations and the limitations of available coding.

Sustained remission was infrequent across all conditions. The highest counts of permanent or sustained remission occurred in asthma (64 individuals; <0.01%), hypertension (12; <0.01%), and diabetes (3; <0.01%), although absolute numbers remained very small. Sustained remission was virtually absent in anaemia, chronic kidney disease, alcohol dependency, and heart failure. Across the cohort, remission typically occurred in a single condition, with only a minority experiencing remission in more than one condition during follow-up.

**Table 2:** Remission of MLTC During Follow-up in the CPRD MLTC Cohort: Condition-Specific Counts of Affected Individuals, Proportions Achieving Remission for ≥ 6 Months, Rates of Permanent or Sustained Remission, and Median Follow-up Time Across 4,745,099 Adults Aged 18 Years and Over in the England

Condition	Number of people with condition	Number of people achieving remission (for ≥ 6 months) (%)	Number of people achieving permanent/sustained remission	Median follow-up (years)
Anaemia	889,562	560,445 (63%)	0	10.4
Alcohol dependency	523,509	1328 (0.25%)	1	7.97
Asthma	1,512,649	676,291 (44.71%)	64	9.54
Chronic kidney disease	1,030,034	1460 (0.14%)	1	12.36
Depression	1,878,060	44,105 (2.35%)	18	8.44
Diabetes	1,942,207	301,313 (15.51%)	3	11.46
Epilepsy	689,874	0 (< 0.01%)	0	10.66
Hypertension	2,508,411	30,124 (1.20%)	12	10.59
Heart failure	1,125,689	1273 (0.11%)	0	9.35
Inflammatory bowel disease	992,264	2 (< 0.01%)	0	11

The number of people with the condition for remission analyses differed from baseline prevalence because remission was evaluated among all individuals who had ever recorded the condition in their clinical history, not solely those with the condition at cohort entry. Consequently, counts for some conditions were lower or higher than their baseline prevalence, depending on whether diagnoses occurred before or after entry into the MLTC cohort.

Baseline prevalence was defined using condition status at cohort entry. Remission analyses used ever-diagnosed denominators to capture conditions diagnosed both before and during follow-up.

Sociodemographic characteristics of individuals who achieved remission are presented in Table 3. Across conditions, the age at remission varied widely: remission tended to occur earlier in conditions with onset in young or mid-adulthood, and later in conditions more closely associated with ageing. Sex distributions also differed markedly by condition. Women represented the majority of remission cases in several conditions, including anaemia and asthma. By contrast, remission was more common among men with alcohol dependency and heart failure. Most remission events occurred among individuals of White ethnicity, reflecting the underlying population structure. The representation of Asian and Black ethnic groups varied by condition, with the highest proportions of remission in these groups observed for diabetes. Remission was observed across all deprivation quintiles, with broadly similar distributions for most conditions. Alcohol dependency demonstrated a strong social gradient, with remission most frequently recorded in the most deprived quintile, whereas remission in hypertension and diabetes showed relatively even distribution across levels of deprivation.

**Table 3:** Sociodemographic Characteristics of Individuals Achieving Remission in the CPRD MLTC Cohort among 4,745,099 Adults Aged 18 Years and Over With Recorded Remission of Long-Term Conditions During Longitudinal Follow-up From England Primary Care Data (1987–2020)

Condition (*n*=)	*n* with remission	Age (mean, SD)	Sex (%)		Ethnicity (%)					Index of Multiple Deprivation				
			Male	Female	White	Asian	Black	Mixed	Other	1 (Least deprived	2	3	4	5 (Most deprived)
Anaemia	560,445	61.89 (17.19)	40.3	59.68	89.1	6.21	3.3	0.47	0.93	18.3	20	19.7	20.2	21.8
Alcohol dependency	1328	48.48 (12.39)	66	34.03	95	2.35	1.59	0.19	0.89	8.78	12.9	18.5	22.5	37.3
Asthma	676,291	53.68 (17.20)	39.7	60.29	91.9	4.23	2.26	0.58	1	17.9	19.1	18.8	20.7	23.5
Chronic kidney disease	1460	62.40 (11.95)	38.6	61.37	94.7	2.67	1.99	0.34	0.27	18	19.5	25.2	19.3	18
Depression	44,105	48.70 (17.08)	36.6	63.36	90.9	5.37	2.15	0.64	0.98	15.2	17.9	18.9	22.7	25.3
Diabetes	301,313	60.05 (13.41)	46.6	53.45	89.4	5.31	3.49	0.49	1.33	19.3	20.7	19.9	20.1	20.1
Epilepsy	0	0	0	0	0	0	0	0	0	0	0	0	0	0
Hypertension	30,124	60.68 (14.93)	40.3	59.66	92.8	3.53	2.42	0.37	0.85	21.4	20.7	19.5	19.1	19.3
Heart failure	1273	58.66 (14.53)	51.9	48.14	92.9	3.26	2.89	0.15	0.76	17.8	21.4	18.1	17.5	25.2
Inflammatory bowel disease	2	36.77 (15.34)	50	50	100	0	0	0	0	50	0	0	50	0

## Discussion

In this large, population-based cohort of adults with MLTC in England primary care, recorded remission of at least one analysed long-term condition was observed during follow-up, although remission varied substantially by condition. The highest recorded remission proportions for at least 6 months were observed for anaemia, asthma, and diabetes, whereas remission of chronic kidney disease, heart failure, inflammatory bowel disease, alcohol dependency, and depression was uncommon, and no remission was documented for epilepsy. Sustained remission was rare across all conditions, and remission most often occurred in a single condition rather than across multiple conditions within the same individual. Patterns of remission varied by age, sex, and ethnicity, but showed relatively little variation across deprivation quintiles.

These findings demonstrate substantial heterogeneity in recorded remission across long-term conditions, reflecting both underlying clinical trajectories and how remission is recorded in routine care. High reported remission rates in asthma likely reflect established coding practices, including the use of “resolved asthma” codes, which are commonly applied after symptom resolution. For conditions subject to sustained monitoring and performance incentives in England primary care, such as those included in the Quality and Outcomes Framework, remission or resolution codes may be applied less frequently, contributing to lower observed remission rates despite clinical improvement. By contrast, sustained remission was exceptionally rare across all conditions, consistent with the strict operational definition applied in this study, which precluded classification as sustained remission if any subsequent prescribing, monitoring, or administrative activity occurred. Differences between baseline prevalence and remission denominators further reflect the use of ever-recorded diagnoses for remission analyses, capturing conditions diagnosed both before and during follow-up. Together, these findings indicate that observed remission patterns in MLTC are influenced not only by clinical change but also by coding behaviours within electronic health records.

The marked variation in recorded remission between conditions likely reflects both differences in underlying clinical course and differences in how remission is identified and coded in routine electronic health records [10, 15, 22]. Higher recorded remission in conditions such as asthma and diabetes may be partly explained by clearer clinical thresholds and more explicit remission or resolution frameworks that are easier to identify in electronic records [10, 16, 17]. By contrast, chronic kidney disease, heart failure, and inflammatory bowel disease are typically characterised by ongoing monitoring, long-term management, and persistent clinical risk, which may make permanent remission less likely to be coded even where disease activity improves [10, 26–28]. Depression and alcohol dependency may also be harder to capture consistently as remission states in routine care because follow-up, relapse prevention, and treatment continuity can vary across clinical settings [29–31]. As a result, differences in recorded remission across conditions likely reflect a combination of disease trajectory, clinical management, and documentation practice [10, 21].

Most remission events occurred in a single condition, consistent with the existing literature, which predominantly examines remission in single-condition cohorts, such as diabetes and asthma, where definitions and minimum durations vary widely. In this framework, remission does not negate the presence of disease across the life course. By contrast, our study focuses on how remission is represented in routine electronic health records and on its implications for measuring current disease burden and care needs. Evidence addressing remission in the context of MLTC remains limited. Population-based studies have largely focused on whether remission signals are recorded in routine data, and scoping work highlights substantial variation in operational definitions. Reports of remission across multiple conditions within the same individual are uncommon and typically confined to specific settings, such as after metabolic or bariatric surgery, and selected populations. These observations align with our findings that multi-condition remission is infrequently documented in routine primary care. These findings highlight that remission, as recorded in routine data, may influence how MLTC burden is quantified and interpreted, with implications for both epidemiological research and clinical care.

This study has several strengths. We used CPRD Aurum, a large and nationally representative primary care dataset linked to hospital records, death registrations, and area-level deprivation indices, enabling comprehensive assessment of remission in routine care. MLTC was defined using a nationally agreed list of long-term conditions, and remission was operationalised using a transparent, condition-specific framework incorporating diagnostic codes, laboratory results, clinical assessments, and treatment patterns. We provide detailed, condition-level estimates and sociodemographic patterns relevant to primary care.

Several limitations should be considered. Remission ascertainment depended on clinical coding, available measurements, and treatment data, and misclassification or under-recording is possible. Although MLTC was defined using 54 long-term conditions, remission could be operationalised for only 10 of these conditions. Our findings, therefore, reflect remission as recorded in a subset of conditions for which EHR-based definitions were feasible and should not be interpreted as covering all conditions contributing to the MLTC burden. Remission definitions could not be applied to all long-term conditions, and differences in follow-up time may have influenced observed remission proportions. Remission events recorded exclusively in secondary care might not always be reflected in primary care records. Some apparent remission events may therefore represent incomplete or inconsistent data capture, changes in coding behaviour, or reduced recording intensity over time, rather than a genuine change in disease status. The study was descriptive and was not designed to assess causal pathways or trajectories of remission across multiple conditions. Because remission was identified only when explicitly recorded or inferable from predefined criteria, estimates should be interpreted as documented remission in primary care records and may underestimate true clinical remission. Our study spans more than three decades (1987–2020), during which there have been substantial changes in electronic health record systems, coding practices, and clinical incentives in England’s primary care. These include the transition from paper to digital records, the introduction of the Quality and Outcomes Framework, and changes in coding systems such as the move towards SNOMED. Such changes may have influenced both the recording of diagnoses and the use of remission or resolution codes over time. As a result, observed patterns of recorded remission may partly reflect temporal variation in coding behaviour and data completeness rather than true changes in disease status. This is particularly relevant for outcomes such as remission, which rely on the absence or presence of specific codes and may be sensitive to changes in documentation practices. Because this variable was derived from routine electronic health records and not independently validated, sex-specific condition estimates should be interpreted cautiously. Recorded remission in routine electronic health records may not reflect clinically meaningful remission for all conditions.

Overall, remission of selected long-term conditions is recorded in England primary care but varies substantially by condition and coding practice. More standardised remission definitions, consistent use of SNOMED codes, and clearer recording of remission onset and duration could improve data quality and support future research examining the role of remission within MLTC care.

### Clinical and policy implications

Our findings indicate that remission is recorded for selected long-term conditions in England’s primary care and can be incorporated into routine management. Our estimates should therefore be interpreted as patterns of documented remission in routine care, rather than direct estimates of true underlying clinical remission. Consistent, condition-specific recording of remission would support accurate registers, recall scheduling, and safety-netting. Incorporating remission status into MLTC and medication reviews could inform treatment de-intensification or continued monitoring where sustained remission is uncommon. Practice and system templates could include remission fields and prompts, with stratified summaries by age, sex, ethnicity, and deprivation to support data quality and equity monitoring. At the commissioning and national levels, standard definitions, shared code lists, and guidance on reconciliation between primary and secondary care would facilitate comparable reporting and enable validation across settings.

## Supplementary Information

Below is the link to the electronic supplementary material.

Supplementary Material 1

## Data Availability

Data may be obtained from a third party and are not publicly available. This study is based on Clinical Practice Research Datalink (CPRD) and is subject to a full license agreement that does not permit data sharing outside of the research team. However, data can be obtained by applying to CPRD (enquiries@cprd.com) for any replication of the study.

## Abbreviations

CPRD: Clinical Practice Research Datalink
EHR: Electronic Health Records
HES: Hospital Episode Statistics
IMD: Index of Multiple Deprivation
ISAC: Independent Scientific Advisory Committee
LTC: Long–Term Condition
MLTC: Multiple Long–Term Conditions
NIHR: National Institute for Health and Care Research
NHS: National Health Service
ONS: Office for National Statistics
QOF: Quality and Outcomes Framework
SD: Standard Deviation
SNOMED: Systematized Nomenclature of Medicine Clinical Terms
UK: United Kingdom
