# Supplementary material for: Remission of long-term conditions among adults with multiple long-term conditions in England primary care: A retrospective cohort study of 4.7 million people
Source: BMC Med. 2026 Aug 10;24:623. doi: 10.1186/s12916-026-05106-1 (PMC13746585; doi:10.1186/s12916-026-05106-1)
Supplement: Supplementary file 1 [file 12916_2026_5106_MOESM1_ESM.docx]

**Remission of long-term conditions among adults with multiple long-term conditions in England primary care: A retrospective cohort study of 4.7 million people**

**Supplementary**:

**Supplementary Table S1.** Published definitions of remission for the 10 analysed long-term conditions, with required remission periods, adapted from scoping review of remission definitions in electronic health records. ^1^

| **Long-Term Condition** | **Explanation of how remission is defined within studies** | **Time Period** |
| --- | --- | --- |
| Anaemia | Normalisation of Hb levels (≥12 g/dl for men and ≥11 g/dl for women) with at least improvement in ≥0.5 g/dl for 3 months | ≥3 months |
| Alcohol dependency | Screening negative (AUDIT-C<5) with at least a 2-point reduction from the previous score | ≥12 months |
| Asthma | No asthma-related event (defined as general practitioner (GP) consultation or admission with a diagnosis code for asthma), no asthma exacerbation, normal lung function and asthma control, no current use of any asthma medication | ≥ 12 months |
| Chronic kidney disease (CKD) | eGFR ≥60 ml/min/1.73 m^2^ | ≥3 months |
| Depression | Patient Health Questionnaire (PHQ)-9 score<5 (following acute treatment) | **≥** 6 months |
| Diabetes | Diabetes HbA1c <6.5% without the use of any anti-diabetic medication | ≥ 6 months |
| Epilepsy | no seizure activity,  having no new antiepileptic drug attempts, and the absence of all seizure-related healthcare events (i.e., seizure-related hospitalisation or seizure-related GP or outpatient visit), QOF data and medical codes indicating a seizure | ≥ 12 months |
| Hypertension | Normal blood pressure (bp) in the absence of antihypertensive drugs. Normal blood pressure varies for age groups:  aged 18-59 years: bp less than 140/90 mm Hg;  aged 60-85 years with diabetes: bp less than 140/90 mm Hg;  aged 60-85 years without diabetes: BP less than 150/90 mm Hg | **≥**6 months |
| Acute heart failure | N-terminal pro-b-type natriuretic peptide (NT-proBNP) level persistently decreased above 1.5 times the baseline level | **≥**6 months |
| Inflammatory bowel disease | *For ulcerative colitis*: a score of ≤ 3 on the Harvey-Bradshaw index or ≤4 on the Ulcerative Colitis Activity Index; endoscopic remission (Mayo Endoscopic Score of 0 or 1) and histologic remission (Simplified Geboes Score <= 0.2); ulcerative colitis endoscopic index of severity (UCEIS) score=0 at the last follow-up and no use of medication | **≥**12 months |
|  | *For Crohn:* Harvey-Bradshaw index or ≤4; Faecal calprotectin (FCP) remission rate (FCP <250mug/g) ; Crohn’s disease activity index (CDAI) < 150 points and no use of medication |  |

**Supplementary Table S2.** READ and SNOMED CT remission and resolution codes identified in CPRD Aurum for the 10 analysed long-term conditions.

| **medcodeid** | **condition** |
| --- | --- |
| 1165041000000115 | Heart failure resolved |
| 12127561000006116 | Recurrent major depressive disorder co-occurrent with anxiety in full remission |
| 12127571000006111 | Recurrent major depressive disorder in partial remission co-occurrent with anxiety |
| 142521000006110 | Major depression single episode, in partial remission |
| 1728531000006116 | Heart failure resolved |
| 1755901000006112 | [X]Single major depressive episode, severe, with psychosis, psychosis in remission |
| 1755911000006110 | [X]Recurrent major depressive episodes, severe, with psychosis, psychosis in remission |
| 182801000006114 | Recurrent major depressive episodes,partial/unspec remission |
| 1975281000006115 | Post-schizophrenic depression, incomplete remission |
| 1975301000006116 | Post-schizophrenic depression, complete remission |
| 2287971000000119 | Diabetes mellitus in remission |
| 2288011000000114 | Type I diabetes mellitus in remission |
| 2288041000000110 | Type 1 diabetes mellitus in remission |
| 2288061000000111 | Type II diabetes mellitus in remission |
| 2288071000000116 | Type 2 diabetes mellitus in remission |
| 2358251000000115 | Alcohol dependence resolved |
| 2404491000000115 | Chronic kidney disease resolved |
| 253531010 | Epilepsy resolved |
| 253532015 | Hypertension resolved |
| 253533013 | Asthma resolved |
| 294621000000118 | Depression resolved |
| 294831019 | Single episode of major depression in full remission |
| 294843018 | Recurrent major depression in full remission |
| 295131012 | Acute alcoholic intoxication in remission, in alcoholism |
| 295142015 | Chronic alcoholism in remission |
| 295254011 | Nondependent alcohol abuse in remission |
| 295320012 | Nondependent antidepressant type drug abuse in remission |
| 296198011 | [X]Recurrent depressive disorder, currently in remission |
| 3033261000006113 | Recurrent major depression in partial remission |
| 3190221000006112 | Major depression in remission |
| 3517985017 | Postpartum major depression in remission |
| 3531371000006112 | Major depression in complete remission |
| 3606411000006115 | Recurrent major depression in remission |
| 459520010 | Diabetes resolved |
| 496491000006117 | Asthma resolved |
| 513711000006118 | Depressed bipolar I disorder in partial remission |
| 513861000006113 | Bipolar affective disorder, currently depressed, in full remission |
| 616801000006114 | Diabetes resolved |
| 647261000006115 | Epilepsy resolved |
| 7060771000006117 | Crohn disease in remission |
| 7502561000006118 | Ulcerative colitis in remission |
| 7515151000006115 | Depressive disorder in remission |
| 790131000006118 | Hypertension resolved |
| 8232571000006118 | Single major depressive episode, severe, with psychosis, psychosis in remission |
| 8232591000006117 | Recurrent major depressive episodes, severe, with psychosis, psychosis in remission |
| 8237831000006115 | Recurrent major depressive episodes, in partial remission |
| 8237841000006113 | Recurrent major depressive episodes, in remission |
| 8237851000006110 | Single major depressive episode, in remission |
| 2993091000006116 | Major depression in partial remission |
| 7060761000006112 | Crohn's disease in remission |

*This table lists only the remission and resolution codes identified in CPRD Aurum. Recorded remission was not determined by these codes alone. For most conditions, remission was classified using a combination of remission/resolution codes (where available), laboratory and clinical measurements, prescribing records, and condition-specific event codes, applied over a required remission period.*

**Supplementary File S3.** READ and SNOMED CT diagnosis code lists used to identify long-term conditions in CPRD Gold and Aurum, 1987–2020

[**CPRD/Medical_Codes/59LTC_CPRD.xlsx at main · big-data-in-health/CPRD · GitHub**](https://github.com/big-data-in-health/CPRD/blob/main/Medical_Codes/59LTC_CPRD.xlsx)

**Supplementary File S4.** CPRD Code Browser user guide (September 2022), describing the dictionary search tool used to identify medical and product codes: [CPRD Code Browser user guide September 2022](https://www.cprd.com/sites/default/files/2022-09/CPRD%20Code%20Browser%20quick%20user%20guide.pdf)

**Supplementary Table S5**: READ and SNOMED CT codes used to identify 54 long-term conditions (LTCs) among adults aged ≥18 years in CPRD Gold and Aurum, 1987–2020 (N = 7,260,829).

Codes were extracted from GP data within the Clinical Practice Research Datalink (CPRD) to define long-term conditions contributing to multimorbidity.

[54 LTC Codes](https://view.officeapps.live.com/op/view.aspx?src=https%3A%2F%2Fraw.githubusercontent.com%2Fbig-data-in-health%2FCPRD%2Frefs%2Fheads%2Fmain%2FMedical_Codes%2F59LTC_CPRD.xlsx&wdOrigin=BROWSELINK)

**Supplementary Table S6**:  Mapping Coverage between READ and SNOMED-CT Concepts for Health Conditions in CPRD.

Mapping completeness was calculated as: 
(Number of READ codes with at least one SNOMED CT match ÷ Total READ codes) × 100%.

|  | **Condition** | **READ codes used** | **SNOMED codes used** | **Successfully mapped (%)** | **Unmapped (%)** |
| --- | --- | --- | --- | --- | --- |
| 1 | Addison’s Disease | 16 | 14 | 93.8% | 6.3% |
| 2 | Anaemia | 5 | 4 | 100% | 0% |
| 3 | Anxiety | 70 | 57 | 93.2% | 6.8% |
| 4 | Depression | 139 | 92 | 100% | 0% |
| 5 | Congenital Heart Disease | 281 | 181 | 100% | 0% |
| 6 | Chromosomal Abnormality | 23 | 13 | 100% | 0% |
| 7 | Bipolar Disorder | 132 | 86 | 100% | 0% |
| 8 | Schizophrenia | 92 | 51 | 99.1% | 0.9% |
| 9 | Chronic Liver Disease | 104 | 77 | 100% | 0% |
| 10 | Aortic Aneurysm | 77 | 45 | 59.8% | 40.2% |
| 11 | Arrhythmia | 331 | 243 | 98.2% | 1.8% |
| 12 | Asthma | 132 | 155 | 100% | 0% |
| 13 | Autism | 13 | 13 | 100% | 0% |
| 14 | Bronchiectasis | 7 | 6 | 100% | 0% |
| 15 | Cancer | 2484 | 2008 | 100% | 0% |
| 16 | Chronic Lyme Disease | 8 | 8 | 75% | 25% |
| 17 | Chronic Pancreatitis | 7 | 7 | 100% | 0% |
| 18 | Chronic Kidney Disease | 41 | 41 | 77.3% | 22.7% |
| 19 | Chronic Pain | 13 | 10 | 100% | 0% |
| 20 | COPD | 100 | 74 | 69.4% | 30.6% |
| 21 | Connective Tissue Disease | 124 | 97 | 100% | 0% |
| 22 | Coronary Heart Disease | 210 | 149 | 95.8% | 4.2% |
| 23 | Heart Failure | 117 | 117 | 98.3% | 1.7% |
| 24 | Hypertension | 108 | 108 | 98.7% | 1.3% |
| 25 | Cystic Fibrosis | 18 | 18 | 100% | 0% |
| 26 | Dementia | 102 | 102 | 100% | 0% |
| 27 | Diabetes | 229 | 229 | 99.1% | 0.9% |
| 28 | Drug or Alcohol Misuse | 407 | 407 | 99.7% | 0.3% |
| 29 | Eating disorders | 31 | 29 | 100% | 0% |
| 30 | Endometriosis | 36 | 36 | 100% | 0% |
| 31 | Epilepsy | 105 | 105 | 98.2% | 1.8% |
| 32 | Gout | 45 | 45 | 98.4% | 1.6% |
| 33 | Hearing Loss | 80 | 58 | 83.3% | 16.7% |
| 34 | HIV/AIDS | 55 | 55 | 97.4% | 2.6% |
| 35 | Inflammatory Bowel Disease | 27 | 27 | 100% | 0% |
| 36 | Long COVID | 4 | 0 | 0% | 0% |
| 37 | Ménière’s Disease | 9 | 9 | 100% | 0% |
| 38 | Multiple Sclerosis | 24 | 24 | 96% | 4% |
| 39 | Musculoskeletal Injury | 458 | 564 | 95% | 5% |
| 40 | Osteoarthritis | 198 | 194 | 100% | 0% |
| 41 | Osteoporosis | 55 | 55 | 100% | 0% |
| 42 | Parkinson’s Disease | 5 | 5 | 100% | 0% |
| 43 | Peptic Ulcer Disease | 139 | 139 | 100% | 0% |
| 44 | Peripheral Neuropathy | 90 | 90 | 100% | 0% |
| 45 | Peripheral Vascular Disease | 245 | 245 | 99.6% | 0.4% |
| 46 | PTSD | 2 | 2 | 100% | 0% |
| 47 | Stroke | 110 | 110 | 99.4% | 0.6% |
| 48 | Thyroid disease | 84 | 84 | 100% | 0% |
| 49 | Transient Ischaemic attack | 14 | 14 | 100% | 0% |
| 50 | Tuberculosis | 200 | 200 | 100% | 0% |
| 51 | Valvular Disease | 365 | 365 | 100% | 0% |
| 52 | Venous Thromboembolism | 58 | 58 | 100% | 0% |
| 53 | Visual Impairment | 58 | 58 | 100% | 0% |
| 54 | Metastatic Cancer | 11 | 11 | 100% | 0% |

**Supplementary Table S7:** Operational algorithms used to classify recorded remission for the 10 analysed long-term conditions.

| **Condition** | **Codeset/source to provide** | **Analysis population / denominator** | **Operational algorithm used to classify recorded remission** | **Required period** |
| --- | --- | --- | --- | --- |
| Anaemia | Anaemia diagnosis codes from Supplementary File S1; remission/resolution codes where available; haemoglobin measurement codes where used. | Individuals with recorded anaemia in the analytic cohort. | Recorded remission was classified where haemoglobin normalised to ≥12 g/dL for men or ≥11 g/dL for women, with improvement of at least 0.5 g/dL from the previous abnormal value. | ≥3 months |
| Alcohol dependency | Alcohol dependency / drug and alcohol misuse diagnosis codes from Supplementary File S1; remission/resolution codes where available; AUDIT-C measurement codes where used. | Individuals with recorded alcohol dependency in the analytic cohort. | Recorded remission was classified where AUDIT-C screening was negative, defined as AUDIT-C <5, with at least a 2-point reduction from the previous score. | ≥12 months |
| Asthma | Asthma diagnosis codes from Supplementary File S1; remission/resolution codes where available; asthma medication codes; asthma event/exacerbation codes; lung-function/asthma-control codes where used; HES/ICD-10 admission codes where used. | Individuals with recorded asthma in the analytic cohort. | Recorded remission was classified where there was no asthma-related event, no asthma exacerbation, normal lung function/asthma control where available, and no current asthma medication use during the remission period. | ≥12 months |
| Chronic kidney disease | CKD diagnosis codes from Supplementary File S1; remission/resolution codes where available; eGFR/renal-function measurement codes where used. | Individuals with recorded chronic kidney disease in the analytic cohort. | Recorded remission was classified where eGFR was ≥60 mL/min/1.73m² for the required remission period. | ≥3 months |
| Depression | Depression diagnosis codes from Supplementary File S1; remission/resolution codes where available; PHQ-9 measurement codes where used; depression treatment codes where used. | Individuals with recorded depression in the analytic cohort. | Recorded remission was classified where PHQ-9 score was <5 following acute treatment, or where relevant remission/resolution evidence was recorded according to the predefined CPRD algorithm. | ≥6 months |
| Type 2 diabetes | Diabetes diagnosis codes from Supplementary File S1; remission/resolution codes where available; HbA1c measurement codes; glucose-lowering medication codes. | Individuals with recorded type 2 diabetes in the analytic cohort. | Recorded remission was classified where HbA1c was <48 mmol/mol (<6.5%) in the absence of glucose-lowering medication during the required remission period. HbA1c values recorded in different units were standardised before applying the threshold. | ≥6 months |
| Epilepsy | Epilepsy diagnosis codes from Supplementary File S1; remission/resolution codes where available; seizure event codes; antiepileptic medication codes; QOF seizure-related codes; HES/ICD-10 seizure-related codes where used. | Individuals with recorded epilepsy in the analytic cohort. | Recorded remission was classified where there was no seizure activity, no new antiepileptic drug attempts, and no seizure-related healthcare events during the remission period. | ≥12 months |
| Hypertension | Hypertension diagnosis codes from Supplementary File S1; remission/resolution codes where available; blood pressure measurement codes; antihypertensive medication codes; diabetes status for threshold selection. | Individuals with recorded hypertension in the analytic cohort. | Recorded remission was classified where blood pressure was normal in the absence of antihypertensive medication. Normal blood pressure was defined as <140/90 mmHg for adults aged 18–59 years; <140/90 mmHg for adults aged 60–85 years with diabetes; and <150/90 mmHg for adults aged 60–85 years without diabetes. | ≥6 months |
| Heart failure | Heart failure diagnosis codes from Supplementary File S1; remission/resolution codes where available; NT-proBNP measurement codes; heart-failure event/admission and treatment codes where used. | Individuals with recorded heart failure in the analytic cohort. | Recorded remission was classified using the predefined NT-proBNP-based algorithm, where NT-proBNP levels persistently improved according to the remission threshold used in the analysis. | ≥6 months |
| Inflammatory bowel disease | IBD diagnosis codes from Supplementary File S1, including Crohn’s disease and ulcerative colitis codes; remission/resolution codes where available; activity-score codes; faecal calprotectin codes; endoscopic/histological remission codes; medication and event/admission codes where used. | Individuals with recorded inflammatory bowel disease in the analytic cohort. | Recorded remission was classified using disease-specific criteria. For ulcerative colitis, remission was based on low activity scores, endoscopic remission, histologic remission, UCEIS score of 0 at last follow-up, and no medication use where required. For Crohn’s disease, remission was based on Harvey-Bradshaw Index criteria, faecal calprotectin <250 µg/g, CDAI <150, and no medication use where required. | ≥12 months |

1.	Hounkpatin H, Barton B, Ogden M, Mathur R, Stuart B, Dambha-Miller H. Understanding Remission of Long-Term Conditions Through Electronic Health Records: Scoping Review. J Med Internet Res. 2026;28:e80796.
